# Mechanisms associated with biogenesis of exosomes in cancer

**DOI:** 10.1186/s12943-019-0963-9

**Published:** 2019-03-30

**Authors:** Kathleen M. McAndrews, Raghu Kalluri

**Affiliations:** Department of Cancer Biology, Metastasis Research Center, University of Texas, MD Anderson Cancer Center, Unit 1906, 1881 East Road, Houston, TX 77054 USA

**Keywords:** Exosomes, Biogenesis, Cancer

## Abstract

Intercellular communication between cellular compartments within the tumor and at distant sites is critical for the development and progression of cancer. Exosomes have emerged as potential regulators of intracellular communication in cancer. Exosomes are nanovesicles released by cells that contain biomolecules and are exchanged between cells. Exchange of exosomes between cells has been implicated in a number of processes critical for tumor progression and consequently altering exosome release is an attractive therapeutic target. Here, we review current understanding as well as gaps in knowledge regarding regulators of exosome release in cancer.

## Background

**Fig. 1:**
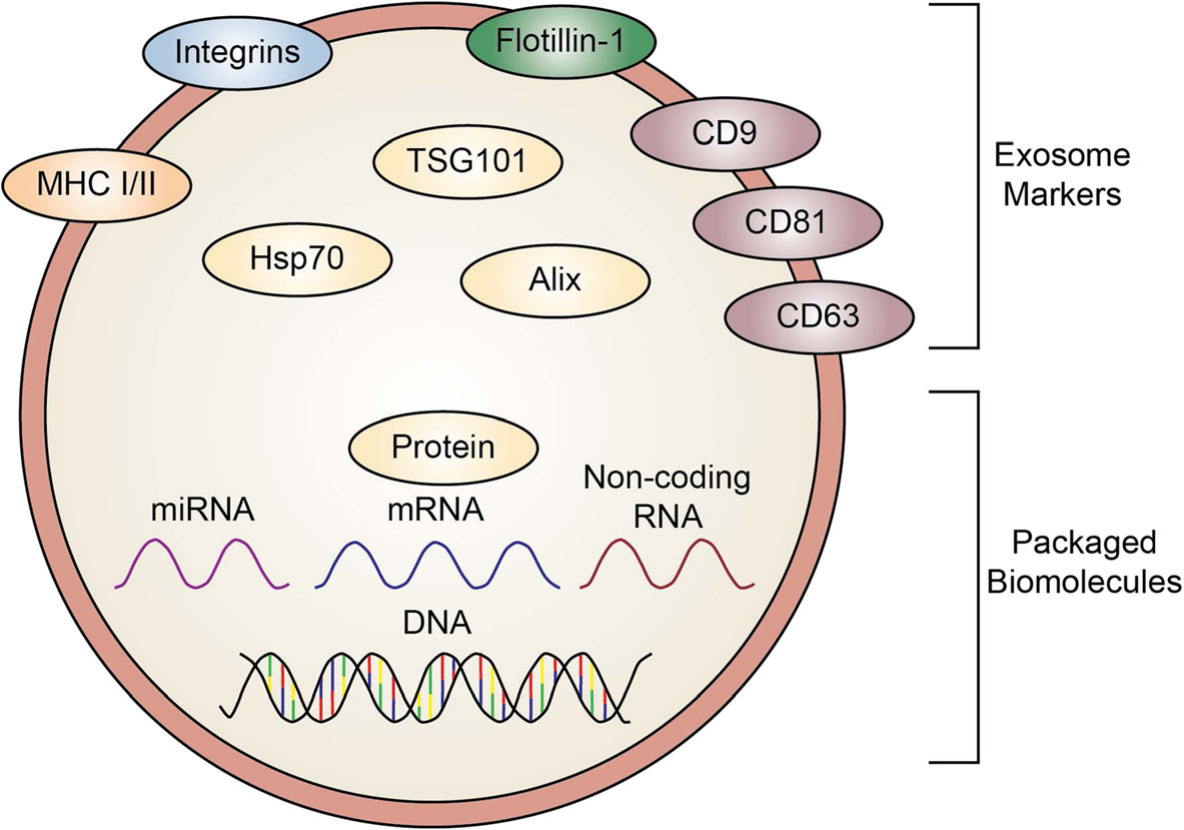
Exosome markers and contents. Common exosome markers include tetraspanins (CD9, CD63, and CD81), flotillin-1, integrins, major histocompatibility complex (MHC) I and II, Hsp70, TSG101, and Alix. Exosomes also contain other proteins, different species of RNA, and DNA

Exosomes have emerged as critical regulators of cell-cell communication. Exosomes are 40–150 nm extracellular vesicles that are generated by all cells and exchanged between cells. Inward budding of the late endosomal membrane encapsulates biomolecules and generates intraluminal vesicles (exosomes) within multivesicular bodies (MVB) [1]. MVBs then fuse with the plasma membrane to release exosomes into the extracellular environment [1]. Exosomes are typically characterized by their size and expression of exosome marker proteins, including CD63, CD81, and CD9 (Fig. 1). Essentially all cell types have been shown to release exosomes in culture [2–14].

Studies have shown ceramide and neutral sphingomyelinase, which converts sphingomyelin into ceramide, is critical for the formation of the intravesicular membrane of MVBs [15]. In dendritic cells, a number of components of the endosomal sorting complex required for transport (ESCRT), including Hrs, signaling transducing adaptor molecule (STAM1), and tumor susceptibility gene 101 (TSG101), are involved in exosome secretion [16]. Syndecan has also been implicated in exosome secretion through its interaction with syntenin, Alix, and several ESCRT proteins [17]. In contrast, cells depleted of ESCRT-0, I, II, and III complexes retained the ability to form MVBs [18], suggesting MVB biogenesis can occur independently of ESCRT in some contexts.

In addition to regulating exosome release, ESCRTs are thought to be involved in packaging of biomolecules into exosomes. ESCRT proteins are involved in packaging of lipids and ubiquitinated proteins into MVBs [19]. Higher-order oligomerization and anchoring of proteins to the plasma membrane is also associated with protein packaging into exosomes [20, 21]. CD63 is involved in ESCRT-independent sorting of premelanosome protein (PMEL) into the intraluminal vesicles of MVBs [22], suggesting there are both ESCRT-dependent and independent pathways of protein sorting in MVBs. However, it is unclear if these MVBs are targeted for degradation in the lysosome or fuse with the cell membrane to release exosomes.

Exosomes are rich in RNA cargo and studies have sought to elucidate the mechanisms regulating RNA loading in exosomes. Many species of RNA are present in exosomes, including microRNA (miRNA), messenger RNA (mRNA), vault RNA, Y-RNA, ribosomal RNA (rRNA) and transfer RNA (tRNA) [23–26]. Preferential accumulation of certain RNA species appears to occur within exosomes [27], suggesting RNA packaging is not random but rather mechanisms exist to package specific RNAs into exosomes. The RNA processing protein Y-box protein 1 has been implicated in packaging of some miRNA [27] and non-coding RNA [26] into exosomes. Heterogeneous nuclear ribonucleoprotein A2B1 (hnRNPA2B1) has also been implicated in miRNA packaging in exosomes through its recognition of miRNA sequence motifs [28]. Breast cancer cell-derived exosomes contain components of the RNA-induced silencing complex (RISC)-loading complex, including Dicer, argonaute-2 (Ago2), and TAR RNA binding protein (TRBP), associated with miRNA [29], which may be an additional mechanism of RNA loading in exosomes. It remains unknown if the aforementioned pathways are broadly applicable to RNA packaging or if additional mechanisms exist to regulate RNA loading in exosomes.

In addition to containing RNA species, exosomes also contain several types of DNA. Mitochondrial DNA (mtDNA) [30–32], single-stranded DNA (ssDNA) [33], and double stranded DNA (dsDNA) [34–36] have been detected in exosomes. DNA incorporated in exosomes can be transferred to and have functional consequences in recipient cells transiently [37]. Exosomal DNA can be transferred to and activate dendritic cells in a stimulator of interferon genes (STING)-dependent manner [38]. While treatment with an epidermal growth factor receptor (EGFR) [39] or topoisomerase-I inhibitors [38] increases DNA packaging into exosomes, the precise mechanisms controlling DNA packaging in exosomes remain to be determined.

Exosomes contain a variety of biomolecules, including DNA, mRNA, miRNA and proteins [40, 41], and can be exchanged between cells. The tumor microenvironment consists of a number of recruited cells that interact to regulate tumor progression and metastasis. As a result, exosomes have emerged as critical regulators of intercellular communication in cancer. Here, we discuss the role of exosomes in cancer and mechanisms controlling their release.

### The function of exosomes in cancer progression and metastasis

**Fig. 2:**
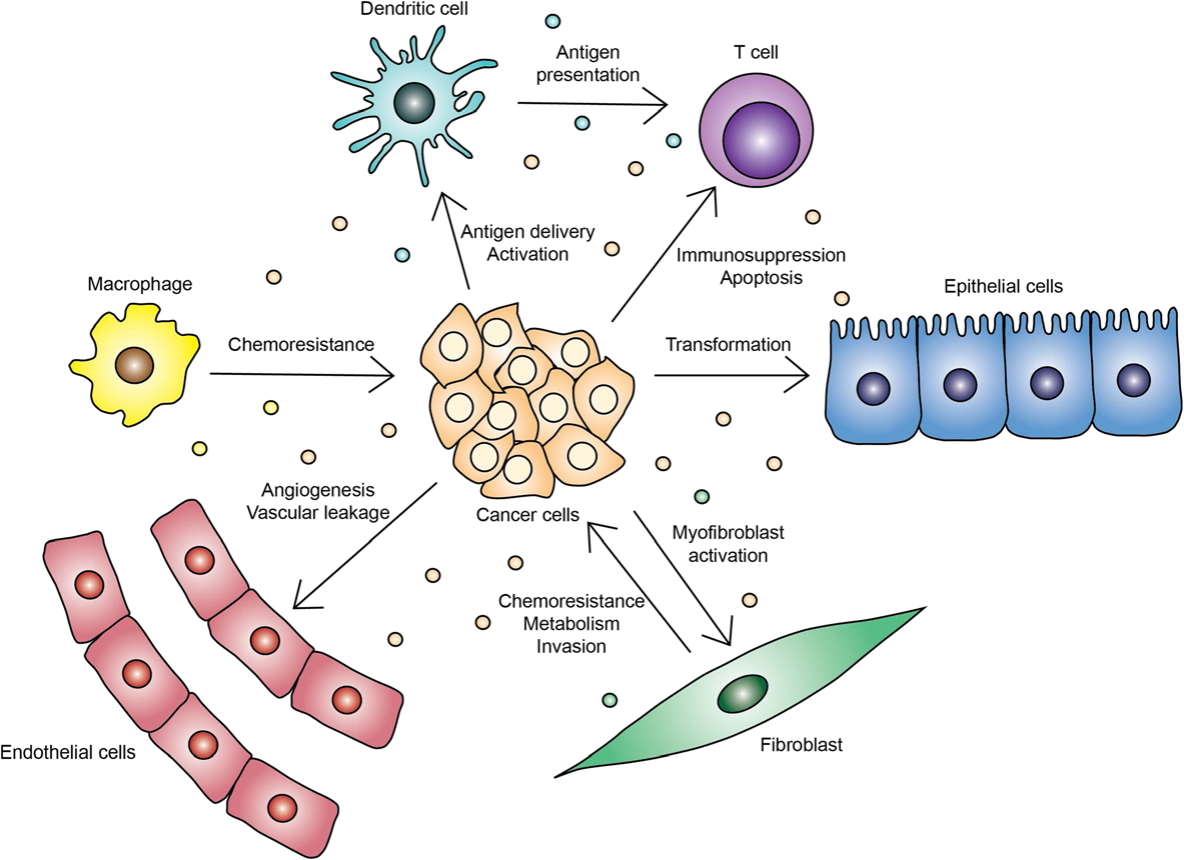
The role of tumor and stromal cell-derived exosomes in cancer. Reported effects of tumor-cell derived exosomes on stromal cells and vice versa within the tumor microenvironment

Tumors have been described as wounds that do not heal due to the chronic inflammatory response observed in tumors [42]. Cancer cells evolve to promote tumor growth and evade immune recognition through intercellular interactions within the tumor microenvironment (Fig. 2). Exosomes derived from breast cancer cells suppress natural killer (NK) cells in vitro [43] and recruit neutrophils to tumors in vivo [44]. Tumor-derived exosomes induce proliferation and expression of STAT3 in myeloid-derived suppressor cells (MDSCs) through Hsp72 [45]. MDSCs are able to inhibit T-cell activation, so exosomes may act to induce immunosuppression through the expansion and activation of MDSCs. Dendritic cell-derived exosomes contain major histocompability complex class I and class II molecules along with T-cell costimulatory molecules, allowing them to function in antigen presentation [46]. Similarly, tumor cell exosomes contain and deliver antigens to dendritic cells for cross-presentation [47]. While these studies suggest tumor cell exosomes can indirectly affect T cell function, tumor exosomes containing Fas ligand can also directly induce CD8^+^ T-cell apoptosis [48]. In addition, PD-L1 is packaged in melanoma, glioblastoma and breast cancer-derived exosomes and is thought to contribute to immunosuppression and lack of response to PD-1 blockade [4, 49, 50]. Collectively, these studies implicate exosomes as mediators of immune regulation in tumors.

The inflammatory tumor stroma is typically also characterized by an accumulation of activated fibroblasts. Interactions between tumor cells and fibroblasts are critical for multiple stages of tumor progression [51]. Tumor cell-derived exosomes initiate fibroblast activation through transfer of transforming growth factor β (TGF-β) [52, 53]. Activated fibroblasts can then reciprocally secrete exosomes containing metabolites which are transferred to cancer cells and reprogram recipient cell metabolism [8]. In addition, fibroblast-derived exosomes can contribute to chemoresistance by increasing the cancer stem cell pool [54] and promote cancer cell invasion through mobilization of Wnt11 [55]. While most studies have reported fibroblast exosomes as being tumor-promoting, in vivo fibroblast subsets are likely to have both tumor-promoting and tumor-suppressive functions [51]; thus, the role of fibroblast exosomes on cancer progression is likely context-dependent. The function of exosomes from other tumor stromal populations is not well characterized, though in pancreatic cancer it has been demonstrated that macrophages transfer miRNA through exosomes to induce gemcitabine resistance, which can be reversed by inhibiting exosome secretion in macrophages [3].

Exosomes have also been implicated as critical regulators of communication between primary tumor cells and distant sites. Exosome secretion is critical for the formation of invadopodia and invasive behavior of breast cancer cells, which may aid in the escape from the primary tumor site [56]. In addition, migratory and invasive behavior can be transferred to non-invasive cells through exosomes [57]. In vivo, uptake of exosomes derived from metastatic cells in cells with lower metastatic capability is associated with transfer of metastatic potential [58]. Inhibition of exosome secretion through knockdown of Rab27A is associated with decreased tumor growth and metastasis in metastatic breast cancer and melanoma models [44, 59]. Rab27A is reported to have functions outside of exosome release, namely in MMP9 secretion [44]; thus, it remains difficult to discern exosome-dependent from exosome-independent effects on tumor progression. Injection of exosomes derived from metastatic cancer cell lines initiates formation of the pre-metastatic niche through recruitment of bone marrow-derived cells and induction of vascular leakage in melanoma, pancreatic cancer, and breast cancer models [5, 59–61]. Integrins in exosomes are also associated with metastatic organotropism, specifically α_6_ is associated with lung metastasis and α_v_ is associated with liver metastasis [5], suggesting exosomal integrins can predict metastatic site. While these studies suggest exogenously provided exosomes are critical for metastasis, it is unclear if they accurately recapitulate native release of exosomes from tumor cells.

### Canonical regulators of exosome secretion: nSMase2 and Rab proteins

Based on the numerous ways exosomes contribute to tumor progression, targeting exosome secretion has emerged as an attractive therapeutic target and has been studied in numerous contexts (Tables 1 and 2). Early studies of exosome release identified ceramide as a regulator of exosome secretion. Ceramide is involved in the inward budding of endosomes to form multivesicular bodies (MVBs) containing exosomes and is generated by neutral sphingomyelinase (nSMase2) [15]. Exosomes are enriched in ceramide and secretion is reduced through inhibition of nSMase2 with siRNA or the small molecule inhibitor GW4869 [15]. Alternatively, treatment of multiple myeloma cells with C6 ceramide induces release of exosomes [62]. A number of other studies have implicated ceramide synthesis in the secretion of exosomes by cancer cells [39, 49, 56, 62–67]. Knockout of nSMase2 reduces angiogenesis and metastasis in breast tumors, which may be mediated through exosome secretion [68]. In addition, mice treated with GW4869 and inoculated with LLC1 cells display a reduced number of lung colonies, likely due to reduced exchange of exosomal miRNAs [69]. GW4869 sensitizes breast tumors to immune checkpoint blockade by reducing secreted exosomal PD-L1 [49]. However, at least one study has reported ceramide as being dispensable for exosome release [70]; consequently, it remains to be determined if this pathway is a conserved regulator of exosome secretion across all cancer types. In addition, it is unclear if the effects of GW4869 in vivo are due to inhibition of exosome release by cancer cells specifically or through organism-wide inhibition of exosome secretion.

**Table 1:** Small molecules and their effect on exosome release in cancer cells

Treatment	Effect on exosome secretion	Cancer cell type
GW4869	Decreases No effect	Bladder cancer cells (T24) [63] Epidermal cancer cells (A431) [39] Liver cancer cells (Huh7) [64] Melanoma cells (B16BL6) [65] Multiple myeloma cells (OPM2) [62] Head and neck squamous cell carcinoma cells (SCC61) [56] Prostate cancer cells (22RV1 and PC3) [66] Head and neck squamous cell carcinoma tumors (mEERL) [67] Breast cancer cells (MDA MB 231) [49] Prostate cancer cells (PC3) [70]
C6 ceramide	Increases	Multiple myeloma cells (OPM2) [62]
Hypoxia	Increases	Breast cancer cells (MCF7, SKBR3 and MDA MB 231) [80]
Shikonin	Decreases	Lung cancer cells (A549) [83]
Acidic pH/ protein pump inhibitors	Increases	Melanoma cells (Mel1-Mel3, Me665/1, MelP1-MelP3 and WM983A) [84, 85]
Tunicamycin	Increases	Cervical cancer cells (HeLa) [90]
Monensin	Increases	Leukemia cells (K562) [94]
Irradiation	Increases	Prostate cancer cells (LNCaP, 22Rv1 and DU145) [97]
UV radiation	Increases	Colon cancer cells (HCT116) [99]
Doxorubicin	Increases	Prostate cancer cells (PC3) [100]
Photodynamic treatment	Increases	Prostate cancer cells (PC3) [100]
Tipifarnib	Decreases	Prostate cancer cells (C4-2B) [106]
Melphalan	Increases	Multiple myeloma cells (SKO-007) [102]
CI-1033/ PF-00299804	Increases	Glioma cells (U373) [39]
Manumycin A	Decreases	Prostate cancer cells (C4-2B) [66]

**Table 2:** Genetic manipulation of exosome release in cancer cells

Gene	Effect on exosome secretion	Cancer cell type
RAB27A	Knockdown decreases	Cervical cancer cells (HeLa) [6] Breast cancer cells (4 T1 [44], TS/A [44], and MDA MB 231 [56,75]) Bladder cancer cells (T24 and FL3) [63] Head and neck squamous cell carcinoma cells (SCC61 [56, 76] and mEERL [67])
RAB27B	Knockdown decreases	Cervical cancer cells (HeLa) [6] Bladder cancer cells (T24 and FL3) [63] Head and neck squamous cell carcinoma cells (mEERL) [67]
PIKfyve	Knockdown increases	Prostate cancer cells (PC3) [74]
Hrs	Knockdown decreases	Head and neck squamous cell carcinoma cells (SCC61) [56]
Syt7	Knockdown decreases	Head and neck squamous cell carcinoma cells (SCC61) [56]
Cortactin	Knockdown increases, overexpression decreases	Head and neck squamous cell carcinoma cells (SCC61) [76]
STAT3	Knockdown decreases	Ovarian cancer cells (OVCAR8) [79]
PKM2	Knockdown decreases	Lung (A549) and cervical cancer cells (HeLa) [83]
Munc13–4	Knockdown decreases	Breast cancer cells (MDA MB 231) [96]
miR-155	Knockdown decreases, overexpression increases	Pancreatic cancer cells (Panc1) [101]
EGFR	Oncogenic EGFRvIII increases	Glioma cells (U373) [39]
Ras	Oncogenic HRas increases	Intestinal epithelial cells (IEC-18) [37, 105]
hnRNP H1	Knockdown decreases	Prostate cancer cells (C4-2B) [66]
Liver Kinase B1	Expression restoration increases	Lung cancer cells (H460 and A549) [107]
EIF3C	Overexpression increases	Liver cancer cells (PLC5, SNU449 and Huh7) [64]

A number of vesicle-trafficking related genes have been implicated in the release of exosomes. In oligodendrocytes, TBC1D10A functions to activate Rab35 in order to induce exosome secretion [71]. Expression of a dominant-negative form of Rab11 in K562 cells is associated with reduced exosome release [72]. Rab11 is also involved in MVB interactions with autophagosomes in K562 cells [72]. Further studies demonstrated Rab11 is involved in the docking of MVBs to the plasma membrane [73]. Upon induction of autophagy, Rab11 colocalizes with the autophagosome marker LC3, which is associated with decreased exosome release [72]. Alternatively, inhibition of PIKfyve, an enzyme that phosphorylates phosphatidylinositol, induces secretory autophagy and increases exosome secretion [74]. Thus, the role of autophagy in the release of exosomes remains to be elucidated and may be context dependent.

**Fig. 3:**
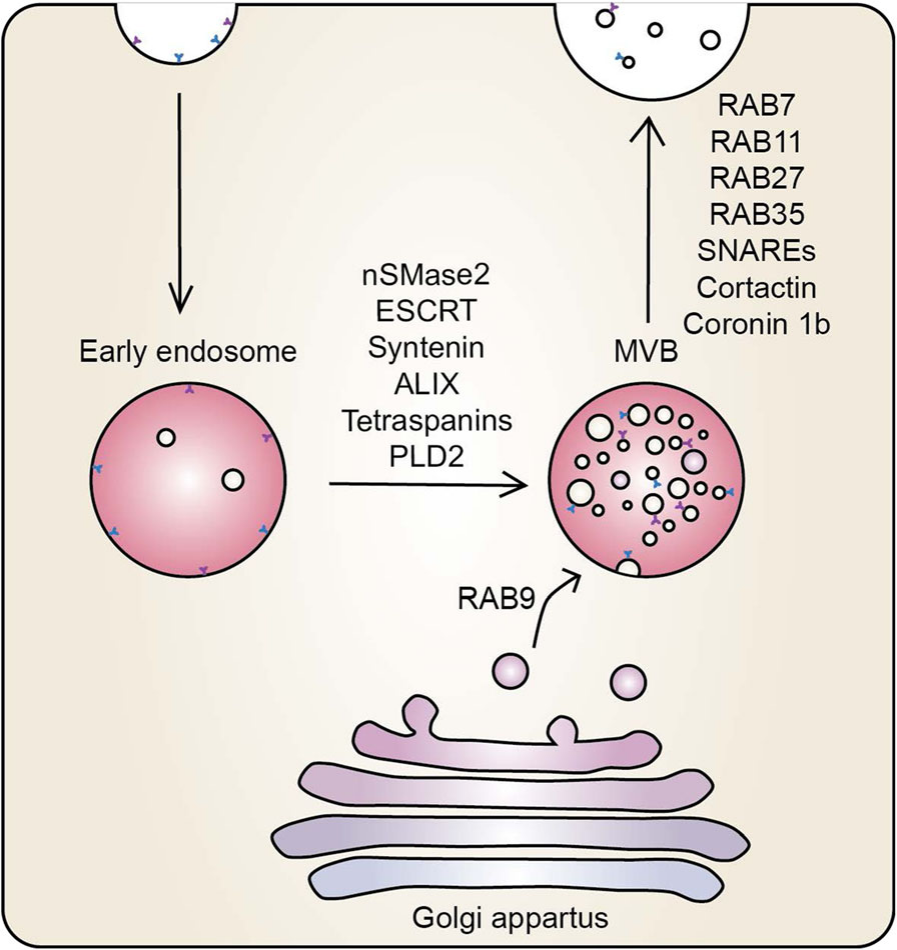
Mechanisms of exosome biogenesis. Multivesicular bodies (MVBs) are formed from budding of early endosomes, which is in part regulated by neutral sphingomyelinase 2 (nSMase2), endosomal sorting complex required for transport (ESCRT), syntenin, ALIX, tetraspanins, and phospholipase D2 (PLD2). In addition, vesicles derived from the Golgi apparatus can fuse with endosomes to be incorporated into MVBs. MVBs fuse with the plasma membrane releasing their contents (exosomes). Membrane docking is regulated by Rab7, Rab11, Rab27, Rab35, soluble NSF attachement protein receptors (SNAREs), cortactin and coronin 1b

While Rab11 appears to be critical for exosome release in K562 cells, it is dispensable for exosome secretion in HeLa cells [6]. In HeLa cells, silencing of Rab2B, Rab5A, Rab9A, Rab27A, and Rab27B reduces exosome secretion, with Rab27A and Rab27B having the largest effects [6]. Rab27A regulates the size of MVBs, whereas Rab27B controls their cellular localization [6]. The role of Rab27A/B in exosome release has been confirmed in many additional cancer cell types [6, 44, 56, 59, 63, 67, 75, 76]. MVBs containing Rab27A are secreted at invadopodia sites [56] and Rab27A in conjunction with cortactin and coronin 1b acts to control stability of MVB docking sites [76] allowing for exosome secretion (Fig. 3). Consequently, Rab27A and exosome secretion are intrinsically linked to cancer cell invasion. In addition, knockdown of Rab27A and Rab27B is associated with increased accumulation of tumor-suppressive miRNA within bladder cancer cells, suggesting the secretion of tumor-suppresive miRNA through exosomes may be critical for tumor progression [63]. Knockdown of Rab27A in metastatic breast cancer cells (4 T1) reduces primary tumor growth and metastasis, but has no effect on nonmetastatic breast cancer (TS/A) [44]. Loss of Rab27A also reduces lung metastasis in melanoma, likely through reducing the recruitment of bone-marrow derived cells in the lung [59].

In addition to regulating tumor cell-intrinsic properties, Rab27A/B are also involved in the exchange of exosomes between different cells within the tumor microenvironment. Genetic deletion of both Rab27A and Rab27B in head and neck squamous cell carcinoma cells reduced exosome-mediated induction of innervation both in vitro and in vivo [67]. Exosome secretion by macrophages is also regulated by Rab27A/B [3]. While the function of Rab27A and Rab27B in exosome release has been established in a number of models, Rab27A has additional exosome-independent roles in tumor progression [44]. In addition, the role of Rab27A/B in exosome secretion is largely based on in vitro experiments, and it remains unclear if Rab27A/B function similarly in vivo.

### Microenvironmental control of exosome release

Cancer cells exist within a complex tumor microenvironment, consisting of recruited endothelial cells, fibroblasts, and immune cells embedded within extracellular matrix that support tumor growth. As the tumor expands, cells compete for nutrients, oxygen, and growth factors; consequently, tumor cells develop mechanisms to survive under these stressful conditions. It has been proposed that tumor cells may use exosome secretion as a way to survive under stress [77, 78].

A hypoxic microenvironment increases the secretion of exosomes by inducing a secretory lysosome phenotype [79]. Exposure of breast cancer cells to hypoxia increases exosome secretion and packaging of hypoxia-related miRNA into exosomes in a hypoxia-inducible factor 1α (HIF-1α)-dependent manner [80]. Exosomes secreted under hypoxic conditions also contained more STAT3 and FAS, which can be transferred to other tumor cells to promote tumor progression and metastasis [79]. Moreover, exosomes from glioblastoma cells cultured in hypoxia induce angiogenesis and tumor growth, potentially through the exchange of hypoxia-related RNAs and proteins [81]. Collectively, these studies demonstrate hypoxia increases secretion of tumor cell-derived exosomes which influence cell behavior in the microenvironment.

Exposure to hypoxia induces downstream metabolic reprogramming to rely on aerobic glycolysis. Pyruvate kinase M2 (PKM2) expression is increased in cancer cells to promote glucose uptake and lactate production through activation of HIF, β-catenin, STAT3, and OCT4-mediated transcription [82]. Studies demonstrated lung cancer cells have high levels of glycolysis, which correlates with high levels of exosome secretion [83]. Inhibition of glycolysis with shikonin decreases exosome release, whereas induction of glycolysis with tumor necrosis factor α (TNF- α) increases exosome secretion [83]. Exosome release can be modulated through the expression of PKM2, suggesting a link between cellular metabolic state and exosome secretion. PKM2 functions to regulate exosome secretion through phosphorylation of synaptosome-associated protein 23 (SNAP-23) [83]. Additional studies demonstrated exosomes are transferred from cancer-associated fibroblasts (CAFs) to modulate cancer cell metabolism to increase glycolysis [8], potentially further modulating exosome secretion.

Hypoxia within tumors is typically associated with increased glycolysis and buildup of lactate in the extracellular environment, which leads to an acidic microenvironment. Intracellular pH also has an effect on the biogenesis of exosomes, with acidic pH (pH = 6.0) increasing exosome secretion [84]. Similarly, inhibition of proton pumps reduces exosome secretion [85]. Alkaline pH reduces exosome secretion as well as exosomal protein and RNA [86]. In addition, acidic extracellular pH has been shown to alter integrin activation. Integrins are critical regulators of exosome uptake [87]; thus, microenvironmental pH may also influence exosome entry into recipient cells. While acidic pH increases exosome release, storage in acidic solutions (pH = 4.0) is associated with exosomal protein degradation [88]. Although this condition is outside of the typical physiological pH range of the tumor microenvironment (pH 6.5–6.9), these studies suggest exosomes may have decreased long-term stability in acidic environments potentially influencing their physiological functions.

Lack of nutrients and dysregulated protein synthesis in cancer cells is also associated with increased protein misfolding and endoplasmic reticulum (ER) stress [89]. Induction of endoplasmic reticulum stress increases MVB formation and subsequent exosome release through ER stress sensors inositol required enzyme 1 (IRE1) and PKR-like ER kinase (PERK) [90]. In choriocarcinoma cells, severe ER stress is associated with secretion of exosomes containing DAMP molecules [91], which may induce an inflammatory response. ER stress also induces splicing of X-box binding protein 1 (XBP1), which is then incorporated in exosomes [92]; thus, ER stress and unfolded protein response may not only influence the secretion of exosomes, but also exosomal packaging of biomolecules.

Calcium signaling plays critical roles in tumorigenesis, progression and metastasis through its involvement in transcription, cell cycle, genotoxicity, angiogenesis and migration [93]. In addition, treatment of cells with monensin, an ionophore that acts as a Na^+^/H^+^ antiporter and reverses the activity of the Na^+^/Ca^2+^ exchanger, increases exosome release [94]. Treatment of cells with thapsigargin, which leads to increased cytosolic Ca^2+^concentration, also increases exosome secretion in neuronal cells [95]. Recently, studies demonstrated Munc13–4 is upregulated in invasive cancer cells and is involved in MVB maturation [96]. Increased Munc13–4 is associated with increased Ca^2+^ uptake and exosome release [96].

In addition to adapting to survive under lack of nutrients and oxygen, tumor cells also acquire the ability to survive after radiation and chemotherapy treatment. Irradiation of prostate cancer cells increases exosome secretion in a p53-dependent manner [97]. Exosomes derived from cells following UV exposure [98] or ionizing radiation [99] are able to elicit a bystander effect in treatment naïve cells through the exchange of RNA species. Treatment with a phototherapeutic or doxorubicin also increased exosome release [100]. Long-term treatment with gemcitabine induces miR-155 expression in pancreatic cancer cells, which is associated with increased exosome secretion and transfer of chemoresistance to surrounding cells [101]. Reduction of exosome secretion through knockdown of miR-155 or Rab27B attenuated these phenotypes [101]. The alkylating agent melphalan induces secretion of exosomes, which are able to stimulate interferon-γ production in NK cells [102]. Thus, DNA damage through radiation and chemotherapy induces release of cancer cell exosomes which have effects on surrounding cells.

### Oncogenic regulation of exosome biogenesis

Studies have demonstrated tumor-bearing patients have increased exosomes in circulation compared to healthy patients [103], suggesting that tumorigenesis is associated with increased exosome secretion. Overexpression of oncogenic EGFRvIII in glioma cells increases secretion of exosomes with EGFRvIII [104]. These vesicles can be transferred to other glioma cells lacking EGFRvIII, resulting in transfer of oncogenic activity [104]. In addition, in cells that are dependent on mutant EGFR, inhibition of EGFR with small molecule inhibitors leads to increased secretion of exosomes with genomic DNA [39]. Consequently, in gliomas driven by EGFR, EGFR is intrinsically linked to the packaging and release of exosomes.

Expression of oncogenic RAS in non-tumorigenic epithelial cells increases exosome secretion [105]. These secreted exosomes have HRAS DNA, RNA, and protein which can be transferred to recipient cells in a transient manner. Similarly, inhibition of RAS signaling with a farnesyl transferase inhibitor (tipifarnib) or manumycin A decreases exosome secretion in prostate cancer cells [106]. Manumycin A-dependent exosome release suppression is associated with inhibition of the oncogenic splicing factor hnRNP H1 in an ERK-dependent manner [66].

In contrast, restoration of liver kinase B1 (LKB1/STK11) expression, a tumor suppressor frequently mutated or lost in lung cancer, increases exosome secretion [107]. Restoration of LKB1 is associated with decreased proliferation but increased cell migration [107]. LKB1 has several functions in nutrient sensing, p53-related pathways [108] and Rab7 interactions [109]; thus, it is unclear which pathways downstream of LKB1 are critical for exosome release.

Eukaryotic translation initiation factors (eIFs), including eIF3, have been implicated in tumorigenesis [110]. In hepatocellular carcinoma (HCC), high expression of eIF3C is associated with poor survival. Exosome secretion is increased in HCC cells expressing eIF3C to promote angiogenesis through S100A11 [64]. Inhibition of eIF3C-dependent exosome release in vitro and in vivo with GW4869 reverses angiogenesis and inhibits tumor growth [64]. Together, these studies implicate oncogenic signaling in the secretion of exosomes.

## Conclusions

There is accumulating evidence that many aspects of tumor progression regulated by cancer cells and the tumor microenvironment can impact the exchange of exosomes. Studies have suggested exosomal cargo can be transferred to recipient cells; however, the fate of exosomes and their cargo in recipient cells remains incompletely understood. Tracking of fluorescently tagged purified exosomes with confocal microscopy demonstrated exosomes enter cells at filipodia, are transferred into endocytic vesicles to the endoplasmic reticulum, and then targeted to lysosomes for degradation in fibroblasts [111]. Other studies showed labeled fibroblast-derived exosomes colocalize with mitochondria in breast cancer cells [31]; thus, exosomes or exosome subpopulations may not be trafficked the same way in all cell types. In addition, it remains to be determined if exosomal cargo is trafficked similarly to the exosomal membrane and membrane-bound proteins. Additional studies could provide critical insight into the fate of exosomes and how this ultimately influences recipient cell behavior.

Most of the studies implicating exosomes in cancer progression utilize in vitro culture systems or inject exosomes isolated ex vivo. As a result, it is unclear if the mechanisms identified from these studies are conserved in vivo. Recently, rat models expressing CD63-GFP were developed to study exosome release in vivo in the whole organism and specifically in neural stem cells [112, 113]. Using a transgenic CD63-GFP mouse model, Manca et al. demonstrated exosomes can be transferred to nursing pups through milk [114]. Disparate results were found by directly nursing mice with endogenously tagged exosomes compared with oral administration of labeled purified exosomes [114], suggesting CD63 may only label a subset of exosomes in vivo or endogenously secreted exosomes have a different uptake pattern compared to purified exosomes. Further characterization of exosome exchange in these models will be critical for understanding the physiological role of exosomes.

In another study, direct exchange of exosomes between cancer cells and host cells was demonstrated using the Cre-LoxP system [58]. Exosomes released from cancer cells entered cells at both local and distant cells; however, the degree of exchange was significantly lower than what was observed in vitro, suggesting the transfer of exosomes in vivo may not be fully recapitulated in vitro. It remains to be determined if the mechanisms of exosome release and entry into recipient cells identified in vitro are also conserved in vivo. Furthermore, while studies have utilized cells genetically engineered to express fluorescently labeled exosomes [29, 115], the use of cell lines precludes studying exosomes in naturally developing tumors and at early stages of tumorigenesis. Additional mouse models to track endogenous exosome release in vivo may clarify the precise mechanisms cancer cells utilize to secrete exosomes and subsequently influence tumor progression.

The small size of exosomes coupled with the lack of techniques to study exosome exchange in distinct cell compartments in vivo has limited our knowledge of the functional role of exosomes in vivo. In addition, while many potential regulators of exosome secretion have been identified, few have been validated in vivo and it is unclear if these regulators are universal to all cell types. The development of additional tools to study exosome exchange between cancer cells, immune cells, fibroblasts, and endothelial cells in vivo will be critical to elucidate interactions within the tumor microenvironment.

The exchange of exosomes within the tumor microenvironment and at distant sites may influence tumor progression, metastasis and therapy response. Unraveling the mechanisms regulating exosome release and fate in recipient cells has the potential to identify novel ways to target intercellular communication and prevent the progression of cancer.

## Abbreviations

Ago2: Argonaute-2
CAF: Cancer-associated fibroblast
DAMP: Damage-associated molecular pattern
dsDNA: Double-stranded DNA
EGFR: Epidermal growth factor receptor
eIF: eukaryotic translation initiation factor
ESCRT: Endosomal sorting complex required for transport
HIF: Hypoxia-inducible factor
hnRNPA2B1: heterogeneous nuclear ribonucleoprotein A2B1
IRE1: Inositol required enzyme 1
LKB1: Liver kinase B1
MDSCs: Myeloid-derived suppressor cells
miRNA: microRNA
mRNA: messenger RNA
mtDNA: mitochondrial DNA
MVB: Multivesicular body
NK: Natural killer
nSMase2: Neutral sphingomyelinase
PERK: PKR-like ER kinase
PKM2: Pyruvate kinase M2
PLD2: Phospholipase D2
PMEL: Premelanosome protein
RISC: RNA-induced silencing complex
rRNA: ribosomal RNA
SNARE: Soluble NSF attachment protein receptor
ssDNA: single-stranded DNA
STAM1: Signaling transducing adaptor molecule 1
STING: Stimulator of interferon genes
TGF-β: Transforming growth factor β
TRBP: TAR RNA binding protein
tRNA: transfer RNA
TSG101: Tumor susceptibility gene 101
XBP1: X-box binding protein 1
